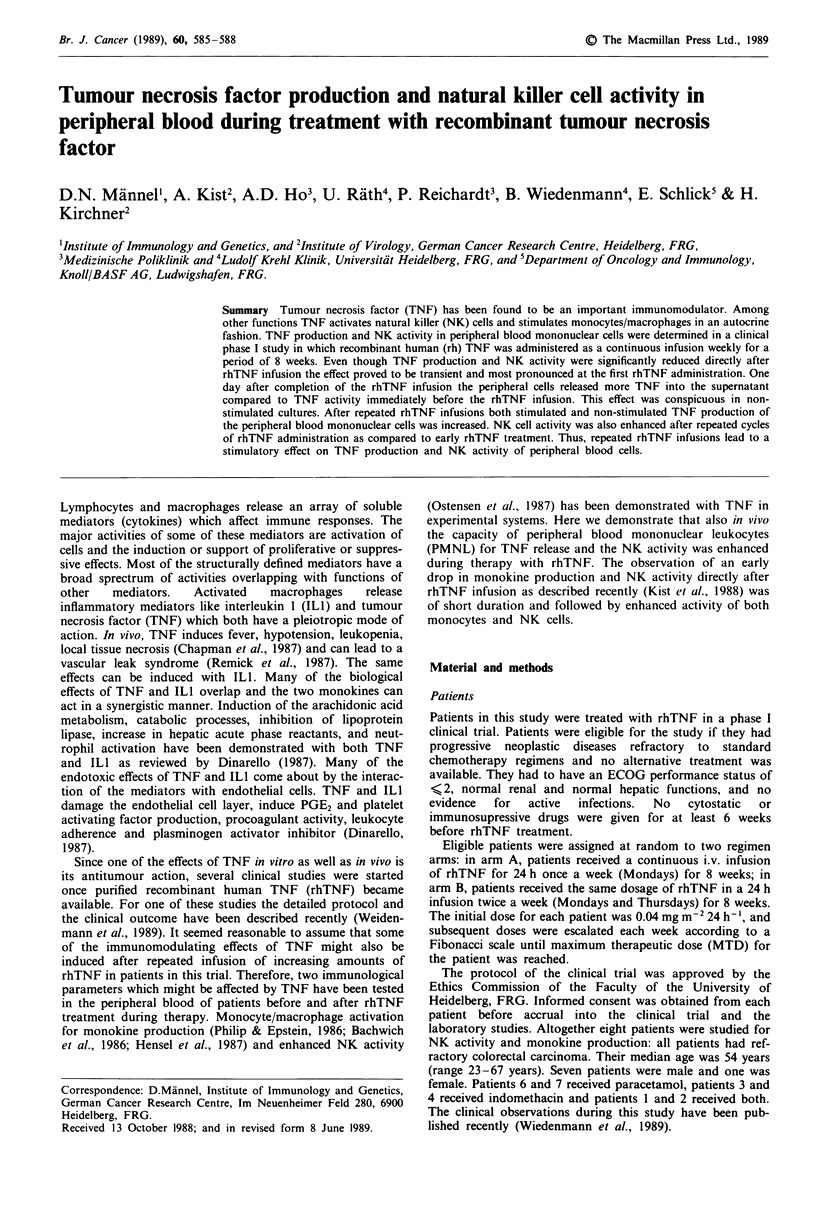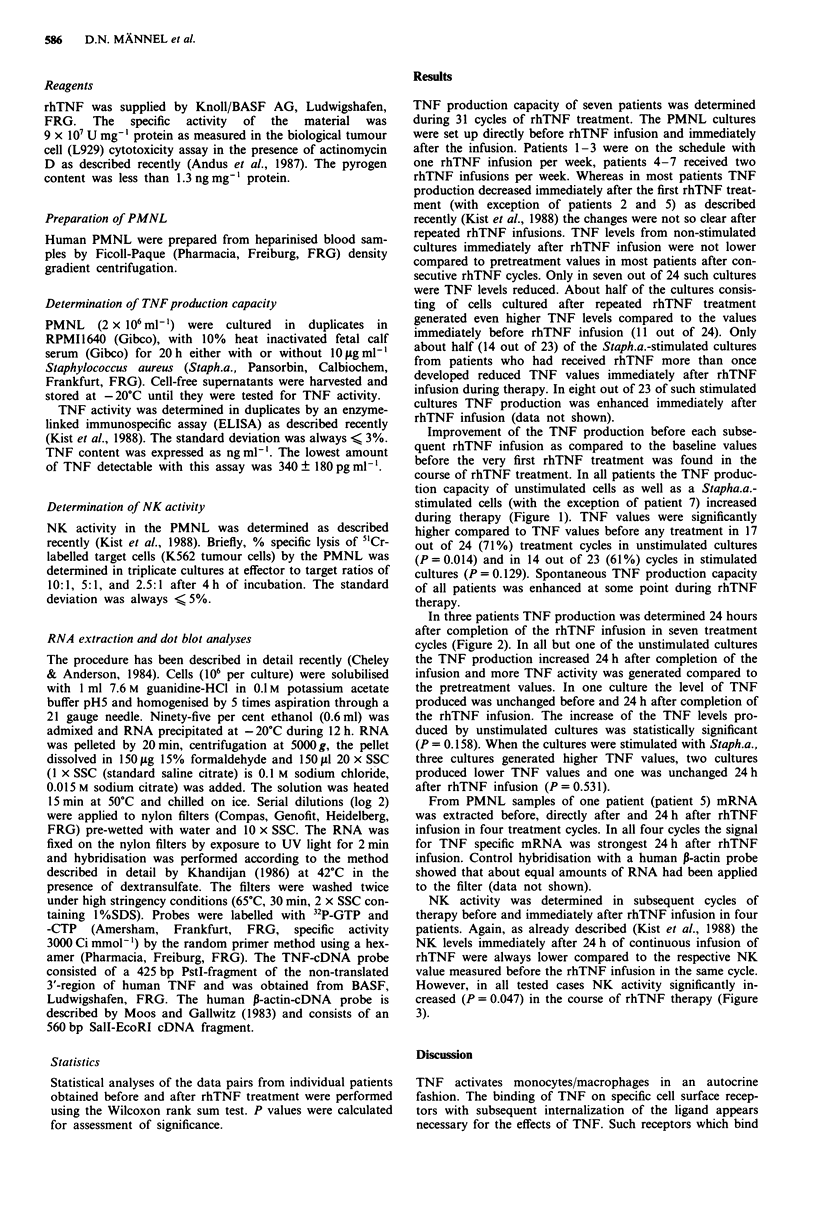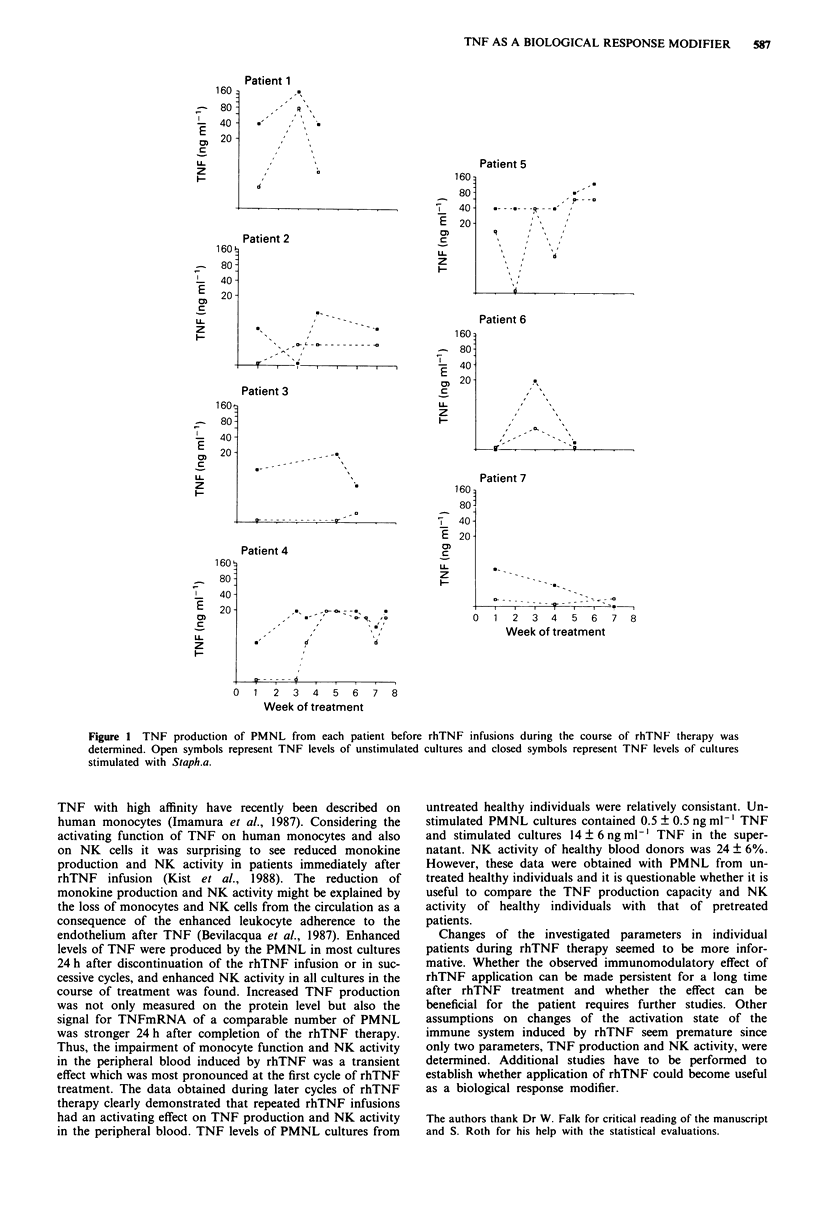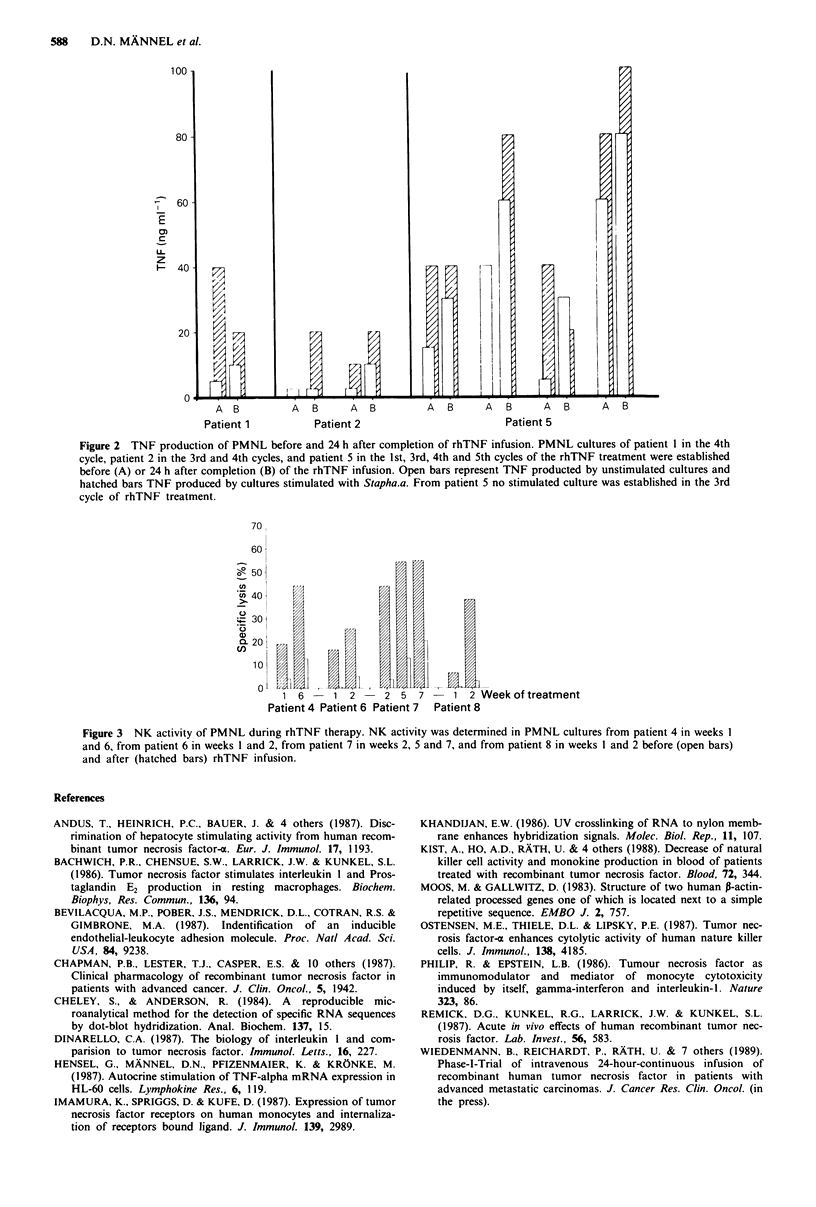# Tumour necrosis factor production and natural killer cell activity in peripheral blood during treatment with recombinant tumour necrosis factor.

**DOI:** 10.1038/bjc.1989.318

**Published:** 1989-10

**Authors:** D. N. MÃ¤nnel, A. Kist, A. D. Ho, U. RÃ¤th, P. Reichardt, B. Wiedenmann, E. Schlick, H. Kirchner

**Affiliations:** Institute of Immunology and Genetics, German Cancer Research Centre, Heidelberg, FRG.

## Abstract

Tumour necrosis factor (TNF) has been found to be an important immunomodulator. Among other functions TNF activates natural killer (NK) cells and stimulates monocytes/macrophages in an autocrine fashion. TNF production and NK activity in peripheral blood mononuclear cells were determined in a clinical phase I study in which recombinant human (rh) TNF was administered as a continuous infusion weekly for a period of 8 weeks. Even though TNF production and NK activity were significantly reduced directly after rhTNF infusion the effect proved to be transient and most pronounced at the first rhTNF administration. One day after completion of the rhTNF infusion the peripheral cells released more TNF into the supernatant compared to TNF activity immediately before the rhTNF infusion. This effect was conspicuous in non-stimulated cultures. After repeated rhTNF infusions both stimulated and non-stimulated TNF production of the peripheral blood mononuclear cells was increased. NK cell activity was also enhanced after repeated cycles of rhTNF administration as compared to early rhTNF treatment. Thus, repeated rhTNF infusions lead to a stimulatory effect on TNF production and NK activity of peripheral blood cells.


Br. J. Cancer (1989), 60, 585-588 © The Macmillan Press Ltd., 1989

### Tumour necrosis factor production and natural killer cell activity in peripheral blood during treatment with recombinant tumour necrosis factor

D.N. Männel[1], A. Kist[2], A.D. Ho[3], U. Räth[4], P. Reichardt[3], B. Wiedenmann[4], E. Schlick[5] & H. Kirchner[2]

[1]*Institute of Immunology and Genetics, and* [2]*Institute of Virology, German Cancer Research Centre, Heidelberg, FRG,* [3]*Medizinische Poliklinik and* [4]*Ludolf Krehl Klinik, Universität Heidelberg, FRG, and* [5]*Department of Oncology and Immunology, Knoll/BASF AG, Ludwigshafen, FRG.*

**Summary** Tumour necrosis factor (TNF) has been found to be an important immunomodulator. Among other functions TNF activates natural killer (NK) cells and stimulates monocytes/macrophages in an autocrine fashion. TNF production and NK activity in peripheral blood mononuclear cells were determined in a clinical phase I study in which recombinant human (rh) TNF was administered as a continuous infusion weekly for a period of 8 weeks. Even though TNF production and NK activity were significantly reduced directly after rhTNF infusion the effect proved to be transient and most pronounced at the first rhTNF administration. One day after completion of the rhTNF infusion the peripheral cells released more TNF into the supernatant compared to TNF activity immediately before the rhTNF infusion. This effect was conspicuous in non-stimulated cultures. After repeated rhTNF infusions both stimulated and non-stimulated TNF production of the peripheral blood mononuclear cells was increased. NK cell activity was also enhanced after repeated cycles of rhTNF administration as compared to early rhTNF treatment. Thus, repeated rhTNF infusions lead to a stimulatory effect on TNF production and NK activity of peripheral blood cells.

Lymphocytes and macrophages release an array of soluble mediators (cytokines) which affect immune responses. The major activities of some of these mediators are activation of cells and the induction or support of proliferative or suppressive effects. Most of the structurally defined mediators have a broad sprectrum of activities overlapping with functions of other mediators. Activated macrophages release inflammatory mediators like interleukin 1 (IL1) and tumour necrosis factor (TNF) which both have a pleiotropic mode of action. *In vivo,* TNF induces fever, hypotension, leukopenia, local tissue necrosis (Chapman *et al.*, 1987) and can lead to a vascular leak syndrome (Remick *et al.*, 1987). The same effects can be induced with IL1. Many of the biological effects of TNF and IL1 overlap and the two monokines can act in a synergistic manner. Induction of the arachidonic acid metabolism, catabolic processes, inhibition of lipoprotein lipase, increase in hepatic acute phase reactants, and neutrophil activation have been demonstrated with both TNF and IL1 as reviewed by Dinarello (1987). Many of the endotoxic effects of TNF and IL1 come about by the interaction of the mediators with endothelial cells. TNF and IL1 damage the endothelial cell layer, induce $PGE_2$ and platelet activating factor production, procoagulant activity, leukocyte adherence and plasminogen activator inhibitor (Dinarello, 1987).

Since one of the effects of TNF *in vitro* as well as *in vivo* is its antitumour action, several clinical studies were started once purified recombinant human TNF (rhTNF) became available. For one of these studies the detailed protocol and the clinical outcome have been described recently (Weidenmann *et al.*, 1989). It seemed reasonable to assume that some of the immunomodulating effects of TNF might also be induced after repeated infusion of increasing amounts of rhTNF in patients in this trial. Therefore, two immunological parameters which might be affected by TNF have been tested in the peripheral blood of patients before and after rhTNF treatment during therapy. Monocyte/macrophage activation for monokine production (Philip & Epstein, 1986; Bachwich *et al.*, 1986; Hensel *et al.*, 1987) and enhanced NK activity (Ostensen *et al.*, 1987) has been demonstrated with TNF in experimental systems. Here we demonstrate that also *in vivo* the capacity of peripheral blood mononuclear leukocytes (PMNL) for TNF release and the NK activity was enhanced during therapy with rhTNF. The observation of an early drop in monokine production and NK activity directly after rhTNF infusion as described recently (Kist *et al.*, 1988) was of short duration and followed by enhanced activity of both monocytes and NK cells.

### Material and methods

### *Patients*

Patients in this study were treated with rhTNF in a phase I clinical trial. Patients were eligible for the study if they had progressive neoplastic diseases refractory to standard chemotherapy regimens and no alternative treatment was available. They had to have an ECOG performance status of $\leqslant 2$, normal renal and normal hepatic functions, and no evidence for active infections. No cytostatic or immunosupressive drugs were given for at least 6 weeks before rhTNF treatment.

Eligible patients were assigned at random to two regimen arms: in arm A, patients received a continuous i.v. infusion of rhTNF for 24 h once a week (Mondays) for 8 weeks; in arm B, patients received the same dosage of rhTNF in a 24 h infusion twice a week (Mondays and Thursdays) for 8 weeks. The initial dose for each patient was $0.04\ mg\ m^{-2}\ 24\ h^{-1}$, and subsequent doses were escalated each week according to a Fibonacci scale until maximum therapeutic dose (MTD) for the patient was reached.

The protocol of the clinical trial was approved by the Ethics Commission of the Faculty of the University of Heidelberg, FRG. Informed consent was obtained from each patient before accrual into the clinical trial and the laboratory studies. Altogether eight patients were studied for NK activity and monokine production: all patients had refractory colorectal carcinoma. Their median age was 54 years (range 23-67 years). Seven patients were male and one was female. Patients 6 and 7 received paracetamol, patients 3 and 4 received indomethacin and patients 1 and 2 received both. The clinical observations during this study have been published recently (Wiedenmann *et al.*, 1989).

Correspondence: D.Männel, Institute of Immunology and Genetics, German Cancer Research Centre, Im Neuenheimer Feld 280, 6900 Heidelberg, FRG.
Received 13 October 1988; and in revised form 8 June 1989.

### Reagents rhTNF was supplied by Knoll/BASF AG, Ludwigshafen, FRG. The specific activity of the material was $9 \times 10^7$ U $mg^{-1}$ protein as measured in the biological tumour cell (L929) cytotoxicity assay in the presence of actinomycin D as described recently (Andus et al., 1987). The pyrogen content was less than 1.3 ng $mg^{-1}$ protein.

### Preparation of PMNL

Human PMNL were prepared from heparinised blood samples by Ficoll-Paque (Pharmacia, Freiburg, FRG) density gradient centrifugation.

### Determination of TNF production capacity

PMNL ($2 \times 10^6$ $ml^{-1}$) were cultured in duplicates in RPMI1640 (Gibco), with 10% heat inactivated fetal calf serum (Gibco) for 20 h either with or without 10 $\mu g$ $ml^{-1}$ *Staphylococcus aureus* (*Staph.a.*, Pansorbin, Calbiochem, Frankfurt, FRG). Cell-free supernatants were harvested and stored at −20°C until they were tested for TNF activity.

TNF activity was determined in duplicates by an enzyme-linked immunospecific assay (ELISA) as described recently (Kist et al., 1988). The standard deviation was always $\leqslant 3\%$. TNF content was expressed as ng $ml^{-1}$. The lowest amount of TNF detectable with this assay was $340 \pm 180$ pg $ml^{-1}$.

### Determination of NK activity

NK activity in the PMNL was determined as described recently (Kist et al., 1988). Briefly, % specific lysis of $^{51}$Cr-labelled target cells (K562 tumour cells) by the PMNL was determined in triplicate cultures at effector to target ratios of 10:1, 5:1, and 2.5:1 after 4 h of incubation. The standard deviation was always $\leqslant 5\%$.

### RNA extraction and dot blot analyses

The procedure has been described in detail recently (Cheley & Anderson, 1984). Cells ($10^6$ per culture) were solubilised with 1 ml 7.6 M guanidine-HCl in 0.1 M potassium acetate buffer pH5 and homogenised by 5 times aspiration through a 21 gauge needle. Ninety-five per cent ethanol (0.6 ml) was admixed and RNA precipitated at −20°C during 12 h. RNA was pelleted by 20 min, centrifugation at 5000 *g*, the pellet dissolved in 150 $\mu$g 15% formaldehyde and 150 $\mu$l 20 × SSC (1 × SSC (standard saline citrate) is 0.1 M sodium chloride, 0.015 M sodium citrate) was added. The solution was heated 15 min at 50°C and chilled on ice. Serial dilutions (log 2) were applied to nylon filters (Compas, Genofit, Heidelberg, FRG) pre-wetted with water and 10 × SSC. The RNA was fixed on the nylon filters by exposure to UV light for 2 min and hybridisation was performed according to the method described in detail by Khandijan (1986) at 42°C in the presence of dextransulfate. The filters were washed twice under high stringency conditions (65°C, 30 min, 2 × SSC containing 1%SDS). Probes were labelled with $^{32}$P-GTP and -CTP (Amersham, Frankfurt, FRG, specific activity 3000 Ci $mmol^{-1}$) by the random primer method using a hexamer (Pharmacia, Freiburg, FRG). The TNF-cDNA probe consisted of a 425 bp PstI-fragment of the non-translated 3′-region of human TNF and was obtained from BASF, Ludwigshafen, FRG. The human $\beta$-actin-cDNA probe is described by Moos and Gallwitz (1983) and consists of an 560 bp SalI-EcoRI cDNA fragment.

### Statistics

Statistical analyses of the data pairs from individual patients obtained before and after rhTNF treatment were performed using the Wilcoxon rank sum test. *P* values were calculated for assessment of significance.

### Results

TNF production capacity of seven patients was determined during 31 cycles of rhTNF treatment. The PMNL cultures were set up directly before rhTNF infusion and immediately after the infusion. Patients 1–3 were on the schedule with one rhTNF infusion per week, patients 4–7 received two rhTNF infusions per week. Whereas in most patients TNF production decreased immediately after the first rhTNF treatment (with exception of patients 2 and 5) as described recently (Kist et al., 1988) the changes were not so clear after repeated rhTNF infusions. TNF levels from non-stimulated cultures immediately after rhTNF infusion were not lower compared to pretreatment values in most patients after consecutive rhTNF cycles. Only in seven out of 24 such cultures were TNF levels reduced. About half of the cultures consisting of cells cultured after repeated rhTNF treatment generated even higher TNF levels compared to the values immediately before rhTNF infusion (11 out of 24). Only about half (14 out of 23) of the *Staph.a.*-stimulated cultures from patients who had received rhTNF more than once developed reduced TNF values immediately after rhTNF infusion during therapy. In eight out of 23 of such stimulated cultures TNF production was enhanced immediately after rhTNF infusion (data not shown).

Improvement of the TNF production before each subsequent rhTNF infusion as compared to the baseline values before the very first rhTNF treatment was found in the course of rhTNF treatment. In all patients the TNF production capacity of unstimulated cells as well as a *Stapha.a.*-stimulated cells (with the exception of patient 7) increased during therapy (Figure 1). TNF values were significantly higher compared to TNF values before any treatment in 17 out of 24 (71%) treatment cycles in unstimulated cultures ($P = 0.014$) and in 14 out of 23 (61%) cycles in stimulated cultures ($P = 0.129$). Spontaneous TNF production capacity of all patients was enhanced at some point during rhTNF therapy.

In three patients TNF production was determined 24 hours after completion of the rhTNF infusion in seven treatment cycles (Figure 2). In all but one of the unstimulated cultures the TNF production increased 24 h after completion of the infusion and more TNF activity was generated compared to the pretreatment values. In one culture the level of TNF produced was unchanged before and 24 h after completion of the rhTNF infusion. The increase of the TNF levels produced by unstimulated cultures was statistically significant ($P = 0.158$). When the cultures were stimulated with *Staph.a.*, three cultures generated higher TNF values, two cultures produced lower TNF values and one was unchanged 24 h after rhTNF infusion ($P = 0.531$).

From PMNL samples of one patient (patient 5) mRNA was extracted before, directly after and 24 h after rhTNF infusion in four treatment cycles. In all four cycles the signal for TNF specific mRNA was strongest 24 h after rhTNF infusion. Control hybridisation with a human $\beta$-actin probe showed that about equal amounts of RNA had been applied to the filter (data not shown).

NK activity was determined in subsequent cycles of therapy before and immediately after rhTNF infusion in four patients. Again, as already described (Kist et al., 1988) the NK levels immediately after 24 h of continuous infusion of rhTNF were always lower compared to the respective NK value measured before the rhTNF infusion in the same cycle. However, in all tested cases NK activity significantly increased ($P = 0.047$) in the course of rhTNF therapy (Figure 3).

### Discussion

TNF activates monocytes/macrophages in an autocrine fashion. The binding of TNF on specific cell surface receptors with subsequent internalization of the ligand appears necessary for the effects of TNF. Such receptors which bind

**Figure 1** TNF production of PMNL from each patient before rhTNF infusions during the course of rhTNF therapy was determined. Open symbols represent TNF levels of unstimulated cultures and closed symbols represent TNF levels of cultures stimulated with *Staph.a.*

TNF with high affinity have recently been described on human monocytes (Imamura *et al.*, 1987). Considering the activating function of TNF on human monocytes and also on NK cells it was surprising to see reduced monokine production and NK activity in patients immediately after rhTNF infusion (Kist *et al.*, 1988). The reduction of monokine production and NK activity might be explained by the loss of monocytes and NK cells from the circulation as a consequence of the enhanced leukocyte adherence to the endothelium after TNF (Bevilacqua *et al.*, 1987). Enhanced levels of TNF were produced by the PMNL in most cultures 24 h after discontinuation of the rhTNF infusion or in successive cycles, and enhanced NK activity in all cultures in the course of treatment was found. Increased TNF production was not only measured on the protein level but also the signal for TNFmRNA of a comparable number of PMNL was stronger 24 h after completion of the rhTNF therapy. Thus, the impairment of monocyte function and NK activity in the peripheral blood induced by rhTNF was a transient effect which was most pronounced at the first cycle of rhTNF treatment. The data obtained during later cycles of rhTNF therapy clearly demonstrated that repeated rhTNF infusions had an activating effect on TNF production and NK activity in the peripheral blood. TNF levels of PMNL cultures from untreated healthy individuals were relatively consistant. Unstimulated PMNL cultures contained $0.5 \pm 0.5$ ng ml$^{-1}$ TNF and stimulated cultures $14 \pm 6$ ng ml$^{-1}$ TNF in the supernatant. NK activity of healthy blood donors was $24 \pm 6\%$. However, these data were obtained with PMNL from untreated healthy individuals and it is questionable whether it is useful to compare the TNF production capacity and NK activity of healthy individuals with that of pretreated patients.

Changes of the investigated parameters in individual patients during rhTNF therapy seemed to be more informative. Whether the observed immunomodulatory effect of rhTNF application can be made persistent for a long time after rhTNF treatment and whether the effect can be beneficial for the patient requires further studies. Other assumptions on changes of the activation state of the immune system induced by rhTNF seem premature since only two parameters, TNF production and NK activity, were determined. Additional studies have to be performed to establish whether application of rhTNF could become useful as a biological response modifier.

The authors thank Dr W. Falk for critical reading of the manuscript and S. Roth for his help with the statistical evaluations.

**Figure 2** TNF production of PMNL before and 24 h after completion of rhTNF infusion. PMNL cultures of patient 1 in the 4th cycle, patient 2 in the 3rd and 4th cycles, and patient 5 in the 1st, 3rd, 4th and 5th cycles of the rhTNF treatment were established before (A) or 24 h after completion (B) of the rhTNF infusion. Open bars represent TNF producted by unstimulated cultures and hatched bars TNF produced by cultures stimulated with *Stapha.a.* From patient 5 no stimulated culture was established in the 3rd cycle of rhTNF treatment.

**Figure 3** NK activity of PMNL during rhTNF therapy. NK activity was determined in PMNL cultures from patient 4 in weeks 1 and 6, from patient 6 in weeks 1 and 2, from patient 7 in weeks 2, 5 and 7, and from patient 8 in weeks 1 and 2 before (open bars) and after (hatched bars) rhTNF infusion.

### References

ANDUS, T., HEINRICH, P.C., BAUER, J. & 4 others (1987). Discrimination of hepatocyte stimulating activity from human recombinant tumor necrosis factor-α. *Eur. J. Immunol.* **17**, 1193.

BACHWICH, P.R., CHENSUE, S.W., LARRICK, J.W. & KUNKEL, S.L. (1986). Tumor necrosis factor stimulates interleukin 1 and Prostaglandin $E_2$ production in resting macrophages. *Biochem. Biophys, Res. Commun.*, **136**, 94.

BEVILACQUA, M.P., POBER, J.S., MENDRICK, D.L., COTRAN, R.S. & GIMBRONE, M.A. (1987). Indentification of an inducible endothelial-leukocyte adhesion molecule. *Proc. Natl Acad. Sci. USA*, **84**, 9238.

CHAPMAN, P.B., LESTER, T.J., CASPER, E.S. & 10 others (1987). Clinical pharmacology of recombinant tumor necrosis factor in patients with advanced cancer. *J. Clin. Oncol.*, **5**, 1942.

CHELEY, S., & ANDERSON, R. (1984). A reproducible microanalytical method for the detection of specific RNA sequences by dot-blot hydridization. Anal. Biochem. **137**, 15.

DINARELLO, C.A. (1987). The biology of interleukin 1 and comparision to tumor necrosis factor. *Immunol. Letts.*, **16**, 227.

HENSEL, G., MÄNNEL, D.N., PFIZENMAIER, K. & KRÖNKE, M. (1987). Autocrine stimulation of TNF-alpha mRNA expression in HL-60 cells. *Lymphokine Res.*, **6**, 119.

IMAMURA, K., SPRIGGS, D. & KUFE, D. (1987). Expression of tumor necrosis factor receptors on human monocytes and internalization of receptors bound ligand. *J. Immunol.* **139**, 2989.

KHANDIJAN, E.W. (1986). UV crosslinking of RNA to nylon membrane enhances hybridization signals. *Molec. Biol. Rep.*, **11**, 107.

KIST, A., HO, A.D., RÄTH, U. & 4 others (1988). Decrease of natural killer cell activity and monokine production in blood of patients treated with recombinant tumor necrosis factor. *Blood*, **72**, 344.

MOOS, M. & GALLWITZ, D. (1983). Structure of two human β-actin-related processed genes one of which is located next to a simple repetitive sequence. *EMBO J.* **2**, 757.

OSTENSEN, M.E., THIELE, D.L. & LIPSKY, P.E. (1987). Tumor necrosis factor-α enhances cytolytic activity of human nature killer cells. *J. Immunol.*, **138**, 4185.

PHILIP, R. & EPSTEIN, L.B. (1986). Tumour necrosis factor as immunomodulator and mediator of monocyte cytotoxicity induced by itself, gamma-interferon and interleukin-1. *Nature* **323**, 86.

REMICK, D.G., KUNKEL, R.G., LARRICK, J.W. & KUNKEL, S.L. (1987). Acute *in vivo* effects of human recombinant tumor necrosis factor. *Lab. Invest.*, **56**, 583.

WIEDENMANN, B., REICHARDT, P., RÄTH, U. & 7 others (1989). Phase-I-Trial of intravenous 24-hour-continuous infusion of recombinant human tumor necrosis factor in patients with advanced metastatic carcinomas. *J. Cancer Res. Clin. Oncol.* (in the press).